# Modelling mammalian implantation *in vitro*

**DOI:** 10.1590/1984-3143-AR2026-0065

**Published:** 2026-09-28

**Authors:** Delanyo Kpeglo, Parisa Noohi, Haidee Tinning, Ella E. Proudly, Rohan M. Lewis, Virginia Pensabene, Niamh Forde

**Affiliations:** 1 Centre for Reproductive Health, University of Edinburgh, Edinburgh, United Kingdom; 2 Discovery and Translational Sciences Department, Leeds Institute of Cardiovascular and Metabolic Medicine, University of Leeds, Leeds, United Kingdom; 3 Faculty of Medicine, University of Southampton, Southampton, United Kingdom; 4 School of Electronic and Electrical Engineering and Pollard Institute, University of Leeds, Leeds, United Kingdom

**Keywords:** embryo implantation, embryo-maternal interactions, species-specific physiology, 3D *in vitro* models, 3D imaging

## Abstract

Embryo implantation is a major reproductive bottleneck, requiring precise spatiotemporal coordination between the embryo and the maternal endometrium. This intricate communication is governed by synchronised molecular signalling, mechanical cues, and endocrine regulation. Although some of these mechanisms are conserved across mammals, marked species differences in the degree of invasiveness of implantation, decidualisation, and placental morphology, as well as in embryonic developmental milestones, limit comparative understanding of early pregnancy across species. Moreover, ethical restrictions on human embryo research, the need to reduce animal use in research, and the oversimplified nature of 2D cultures, which cannot replicate the 3D, multicellular organisation of the implantation site, have further constrained progress. Recent advances in 3D *in vitro* technologies, including organoids, blastoids, and biomaterial-based cultures, provide more physiologically relevant platforms for studying embryo–endometrium interactions. When integrated with organ-on-a-chip systems, these models enable dynamic control of hormonal gradients, paracrine signalling, and fluid flow, closely mimicking the native implantation microenvironment, unlike static cultures. These systems have yielded key insights into epithelial–stromal crosstalk, immune regulation, and vascular remodelling at the maternal-foetal interface. However, challenges remain regarding reproducibility and rigorous biological validation. This review explores the biological principles of implantation across mammalian species. It evaluates *in vivo* animal models and 2D and 3D *in vitro* models for their ability to recapitulate species-specific implantation strategies. Furthermore, we highlight how integrating cellular diversity, biomechanical forces, and species-specific tissue architecture into bioengineered 3D platforms, together with advanced imaging, can bridge the gap between *in vitro* approaches and complex *in vivo* physiology. The convergence of these technologies is poised to transform implantation research and ultimately improve reproductive outcomes.

## Introduction

Embryo implantation in mammals is a highly coordinated process orchestrated by the developing embryo and the endometrium, and it is a crucial bottleneck for successful pregnancy. Embryo mortality during the peri-implantation period contributes to reproductive failure (Muter et al., 2023; Perkel et al., 2015). In humans, early pregnancy loss during this period is estimated at 30-40% of all conceptions (Macklon et al., 2002; Wilcox et al., 1988). Similarly, in sheep and cattle, 20-40% of viable embryos are lost during the critical maternal recognition of pregnancy (Diskin and Morris, 2008). In pigs, embryonic mortality ranges from 30-50%, driven by limited uterine space and the complex dynamics of conceptus elongation (Geisert and Schmitt, 2002). Conversely, in laboratory rodents, this is about 10-15% (Drews et al., 2020). These stark interspecies variations highlight the need to develop species-specific models to fully understand the mechanisms underlying early pregnancy failure.

The timing of implantation varies among mammals, but it generally begins when the embryo attaches to the luminal epithelium of the uterine endometrium. In domestic species, including pigs and ruminants (such as cattle, sheep, and goats), implantation is a superficial process whereby the elongated, filamentous conceptus attaches to the endometrium without invading into the underlying stromal compartment (Amelkina et al., 2025; Johnson et al., 2025) (Figure 1). In humans, the hatched blastocyst invades the endometrium and becomes fully embedded within the stroma, followed by deep invasion of extravillous trophoblasts (EVTs) into the underlying myometrium (interstitial implantation). Meanwhile, the stroma undergoes decidualisation, forming a nutrient-rich decidua to support early pregnancy (Amelkina et al., 2025). Decidualisation is a highly diverse process that prepares the uterine lining for embryo attachment, but it occurs only in certain mammalian species. While it occurs spontaneously in humans and select primates, in other species, such as mice, it is strictly induced by the implanting embryo. In contrast, in mice, although similar to humans, the process involves eccentric implantation, where the embryo adheres to the luminal epithelium, which then closes over it, but the embryo does not invade deeply into the stromal tissue (Bondarenko et al., 2023).

**Figure 1:**
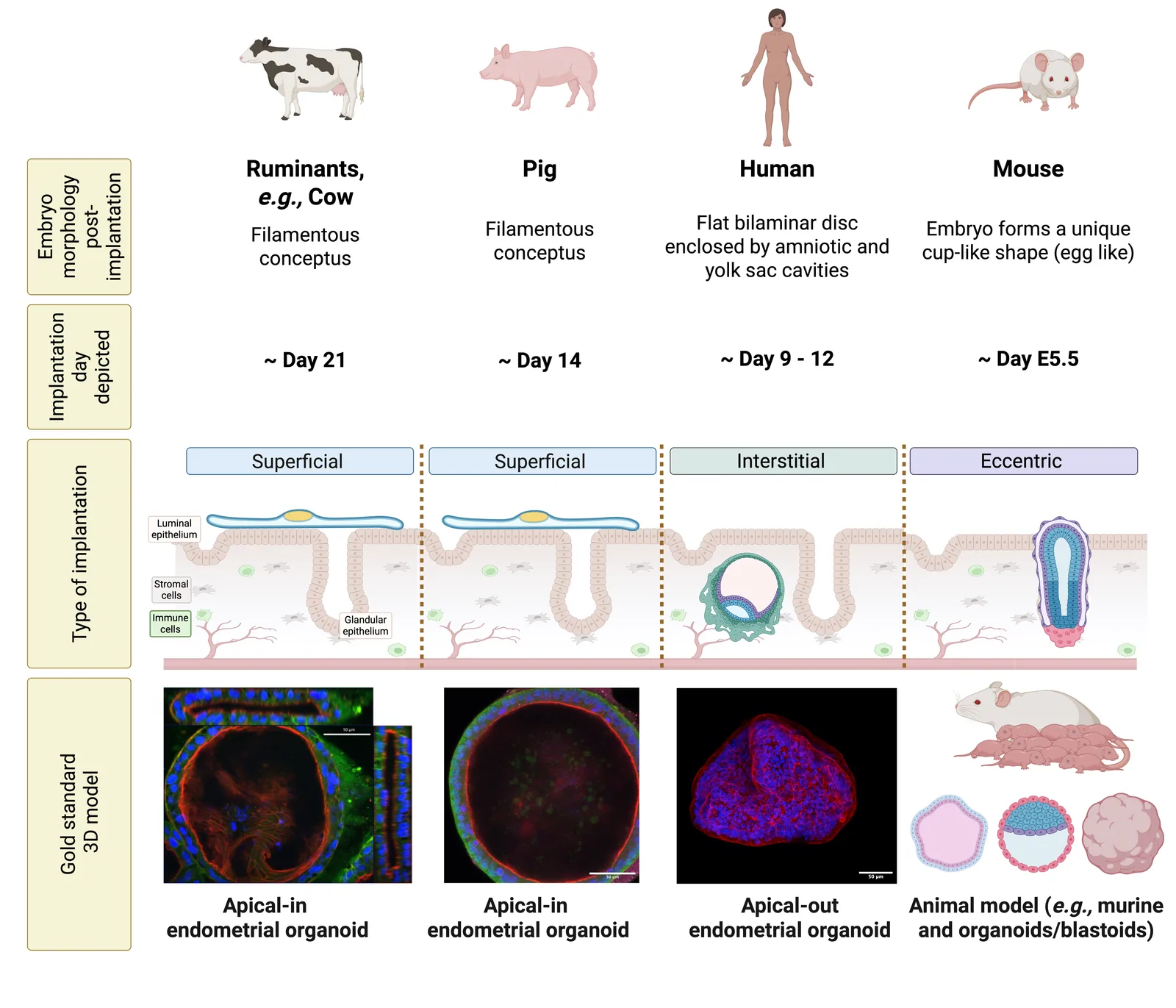
A comparison of post-implantation embryo morphology, implantation day and invasion, and models currently in use to study endometrial-embryo interactions. Bovine apical-in endometrial organoid taken from Edge et al. (2026), porcine endometrial organoid taken from Edge et al. (2026), and human apical-in endometrial organoid taken (Lewis et al., unpublished).

Irrespective of the species, successful implantation depends on accurate timing and spatial coordination between a developmentally competent embryo and a receptive endometrium, which together ensure attachment and, in humans, invasion of maternal tissue (Cha et al., 2012; Wang et al., 2023). If this crosstalk is not synchronised, recurrent implantation failure and early pregnancy loss can occur. However, our understanding of the mechanisms underlying successful implantation, and therefore those that cause implantation failure, remains limited. This knowledge gap highlights the inherent difficulty of studying the peri-implantation period, in which ethical restrictions and limited access to tissues from specific species (e.g. humans) hinder direct investigation of embryo implantation mechanisms (Dimova et al., 2025; Horsley et al., 2025). Historically, much of what is known about implantation has been derived from mouse models, owing to their genetic tractability and short gestation (Altmäe et al., 2012; Zhang et al., 2013). While understanding this process in food production species has been possible using *in vivo* models, which have provided invaluable insights, until recently there have been limitations in understanding the mechanism of implantation due to the lack of tools available for, *e.g.,* mouse models. Moreover, key species-specific differences in endometrial physiology, implantation dynamics, placental architecture, timing of genome activation, embryo morphology, decidualisation, and pregnancy duration limit their translational relevance across placental mammals (Niakan et al., 2012). To address these limitations, two-dimensional (2D) cultures of endometrial and trophoblast cells have enabled the study of specific molecular crosstalk between the embryo and the surrounding endometrial environment (Weimar et al., 2013). However, these platforms fail to capture the three-dimensional (3D) architecture, intricate cell-cell interactions, co-culture effects, biomechanical cues with fluid flow, and the cell-extracellular matrix (ECM) interactions, all of which occur *in vivo* (Kim et al., 2025; Molè et al., 2026).

Although *in vivo* models continue to provide insights into key aspects of implantation and pregnancy, the limitations of animal models and 2D cultures, together with advances in stem cell biology, biomaterials, and tissue engineering, have led to the development of more physiologically relevant 3D *in vitro* implantation platforms. These include stem cell-derived embryo-like structures (blastoids), endometrial and trophoblast organoids, scaffold-based cultures, and sophisticated microfluidic cultures or organ-on-a-chip platforms, all of which address the ethical and practical constraints of studying human embryo implantation (Harrison and Bailey-Hytholt, 2025). Moreover, the development of bovine and porcine organoids has become a valuable tool for investigating livestock physiology and exploring aspects of mammalian implantation. This enables the study of species-specific attachment and signalling mechanisms, such as interferon tau (IFNτ) dynamics in cattle, which differ significantly from human Chorionic Gonadotropin (hCG) signalling during invasive implantation (Edge et al., 2026). By recapitulating the spatial organisation, ECM composition, and dynamic signalling environment unique to each species, these 3D *in vitro* platforms offer opportunities to investigate implantation-related processes, including epithelial remodelling, stromal decidualisation, and trophoblast invasion, as well as hormonal responsiveness.

In this review, we explore the biological principles underlying mammalian implantation, discuss what 3D *in vitro* models encompass, evaluate the insights that existing 3D *in vitro* cultures or models have provided into implantation biology, and highlight the technical and conceptual challenges in developing a 3D *in vitro* implantation model, including scaffold selection and imaging techniques. It is important to continually improve 3D *in vitro* models of implantation, so assessing these emerging tools is essential for defining their role and guiding the development of next-generation 3D implantation models.

## Mammalian embryo implantation

In mammals, implantation is driven by reciprocal signalling between the embryonic trophoblast cells and the uterine endometrium (Wang and Dey, 2006). The trophectoderm forms the primary interface with the endometrium and rapidly differentiates and functionally specialises in response to endometrial cues (Johnson et al., 2025; Knöfler et al., 2019). Trophoblast cells secrete cytokines, growth factors, and extracellular vesicles that, together with the circulating hormonal milieu, influence endometrial receptivity, immune tolerance, and tissue remodelling (Karimi et al., 2025). Although aspects of embryo-endometrial communication are conserved across mammals, the cellular dynamics, timing, and depth of implantation vary considerably between species (Gellersen and Brosens, 2014). Understanding these differences is vital for benchmarking and developing physiologically relevant *in vitro* models.

Embryo implantation involves three stages: apposition, adhesion, and invasion (Kim and Kim, 2017). During apposition, the embryo forms reversible contact with the luminal epithelium. This transient stage allows the selection of developmentally competent embryos before stable attachment and is characterised by changes in epithelial polarity and glycocalyx composition, as well as protrusions such as pinopodes (Huang et al., 2023). Adhesion is a crucial step in transitioning from transient to stable attachment of the embryo to the endometrium. In species with invasive implantation (*e.g.*, mice and humans), this process is mediated by cell adhesion molecules, including integrins, cadherins, and selectins, which are expressed on trophoblast and epithelial cells (Cha et al., 2012; Huang et al., 2023). This, in turn, triggers local remodelling of epithelial junctions and the basement membrane, facilitating subsequent interactions with the underlying stromal tissue compartment (Huang et al., 2023). The process of invasion is the most divergent among mammals. In humans and mice, the luminal epithelium at the implantation site undergoes apoptosis, allowing the trophoblast to breach the epithelial barrier and migrate into the underlying stromal tissue (Bondarenko et al., 2023; Muter et al., 2023). In contrast, in pigs and ruminants, including cows, sheep, and goats, implantation is non-invasive, with the conceptus remaining in contact with the epithelial surface without breaching the basement membrane (Johnson et al., 2025). In pigs, attachment is mainly mediated by focal adhesions and paracrine signalling, resulting in an epitheliochorial placenta in which epithelial integrity is maintained (Johnson et al., 2025). In ruminants such as cows, specialised binucleate trophoblast cells fuse with uterine epithelial cells to form a temporary syncytiotrophoblast, allowing molecular exchange while preserving the overall epithelial structure (Amelkina et al., 2025; Davenport et al., 2023; Johnson et al., 2025). In invasive species such as mice and humans, trophoblast differentiation supports significant remodelling of the extracellular matrix and interactions with the maternal immune and vascular systems, whereas in species with non-invasive implantation, trophoblast–epithelial communication is more predominant (Griffith and Wagner, 2017; Karimi et al., 2025). There are differences among species with similar implantation types. For example, compared to mice, human trophoblast invasion is deeper and lasts longer, and decidualisation occurs independently of the presence of the embryo. Furthermore, the human endometrium exhibits considerable variation in receptivity and responsiveness among individuals, influenced by genetic, environmental, and clinical factors. These features emphasise the importance of human or species-specific experimental systems that mirror the cellular diversity and dynamic regulation of implantation within their native environment niche (Jabri et al., 2025).

The maternal endometrium consists of a heterogeneous cell population that coordinates to prepare the uterine endometrium for the structural and biochemical conditions required for embryo attachment. Among these, luminal and glandular epithelial cells serve as the primary sensors for embryonic contact. In all mammals studied, including sheep, cow, and mouse, epithelial cells provide essential histotrophic support by secreting cytokines and growth factors, including leukaemia inhibitory factor (LIF), insulin-like growth factors (IGFs), interleukins 6 and 11, and platelet-derived growth factor (PDGF) (Gellersen and Brosens, 2014; Wang and Dey, 2006). These signalling molecules facilitate communication between the uterine endometrium and the embryo, thereby supporting embryonic development. Simultaneously, in some species, stromal fibroblasts undergo decidualisation, a process that is spontaneous in humans and is driven by progesterone and the cyclic adenosine monophosphate (cAMP) signalling pathway, which reprogrammes the stroma into a selective biosensor of embryo quality (Gellersen et al., 2007; Gellersen and Brosens, 2014).

During decidualisation, stromal cells undergo a significant phenotypic transformation and secrete factors such as prolactin and insulin-like growth factor binding protein 1 (IGFBP-1), which facilitate targeted remodelling of the ECM. The resulting specialised phenotype creates a permissive yet selective environment that acts as a biosensor of embryo quality, supporting early placentation while restricting the development of incompetent embryos (Gellersen et al., 2007; Gellersen and Brosens, 2014). Mechanistically, this sensing is driven by decidualised endometrial stromal cells in close coordination with the overlying luminal epithelium. Rather than merely assessing cell viability, this maternal sensor dynamically evaluates the embryo's metabolic fitness and chromosomal competence by monitoring blastocyst secretions, including hCG and serine proteases such as trypsin. While developmentally competent embryos trigger a supportive, migratory response in the decidua, chromosomally abnormal, morphologically arrested, or incompetent embryos induce a maternal cellular stress response, prompting the stroma to selectively withdraw key implantation factors and actively reject the embryo.

Within this microenvironment, a sophisticated immune dialogue is established. Uterine natural killer (uNK) cells, macrophages, and regulatory T cells are key orchestrators of maternal immune tolerance and trophoblast invasion (Moffett and Colucci, 2014). These immune populations, particularly uNK cells, are essential for the physiological remodelling of maternal spiral arteries. Through endothelial cell-mediated angiogenesis, this vascular remodelling supports the high-volume nutrient exchange required for advancing gestation, thereby highlighting the remarkable cellular complexity of the human implantation environment (Knofler et al., 2019; Smith, 2001).

At the molecular level, implantation is governed by a complex network of cytokines, chemokines, and morphogens organised into carefully regulated spatial and temporal gradients (Vilella et al., 2015). These biochemical signals integrate with biophysical cues, including ECM stiffness, cellular traction forces, and mechanical feedback, to regulate trophoblast migration and invasion depth (Altmae et al., 2017). Importantly, these parameters differ significantly between species. The patterns of stromal decidualisation, immune cell recruitment, and vascular remodelling highlight the need for an experimental model that recapitulates both the biochemical and biomechanical features of human implantation or the species in question.

Endometrial receptivity is closely regulated in all mammals by the ovarian steroids, oestrogen and progesterone, which coordinate cyclical changes in gene expression, cellular function, and tissue structure. The window of implantation is a brief period during which the endometrium can support embryo attachment, and disruption of hormonal signalling and timing can lead to implantation failure even with a viable embryo (Karimi et al., 2025). In mice and humans, this window is characterised by extensive transcriptional and epigenetic reprogramming of the epithelial and stromal compartments (Altmae et al., 2017). In contrast, in pigs and ruminants, maternal recognition of pregnancy relies on conceptus-derived signals, such as IFNτ, which prevent luteolysis and thereby maintain progesterone levels (Davenport et al., 2023; Johnson et al., 2025).

Together, these findings highlight species-specific differences in implantation strategies and regulatory mechanisms, emphasising the need to select models that align with both the biological question and the implantation stage under investigation. These complexities underscore the need for advanced 3D *in vitro* systems, such as organoids and assembloids, that more accurately recapitulate the unique human or species-specific implantation microenvironment.

## Conventional models of embryo implantation

Studies of embryo implantation have traditionally relied on animal models and simplified 2D *in vitro* cultures. These approaches have helped define the fundamentals of implantation but capture only a subset of the cellular and physiological features, especially those unique to human implantation. A critical evaluation of the strengths and limitations of these models is therefore essential to contextualise the insights they have provided and to motivate the development of more representative and physiologically relevant 3D *in vitro* systems (Edge et al., 2026; Mole et al., 2026; Wang and Dey, 2006).

2D *in vitro* cultures of endometrial epithelial or stromal cells have been widely used to investigate aspects of hormone signalling and gene regulation under highly controlled conditions (Gellersen et al., 2007). These 2D co-cultures, in which trophoblast cells are combined with endometrial monolayers, have further enabled the study of adhesion molecules and early attachment events (Aplin and Kimber, 2004). However, cells grown on rigid 2D plastic substrates can show altered cell polarity, differentiation, and gene expression compared with their *in vivo* counterparts (Vilella et al., 2015). These models offer scalability and experimental simplicity, but they fail to capture spatial gradients, the arrangement of cells and tissue layers, ECM interactions, or the biomechanical context of the implantation niche in humans (Rawlings et al., 2021) or in species such as mice, cows, or pigs.

*In vivo* analysis of implantation remains the gold standard to understand the mechanisms of implantation in mammals. The mouse has been the primary model, providing detailed insights into hormonal regulation and embryo-uterine signalling (Wang and Dey, 2006) and enabling precise temporal analysis of key implantation events (Bondarenko et al., 2023; Wang and Dey, 2006). However, significant interspecies differences among mammals (discussed above) can limit the direct translation of these findings to other species, including humans. To understand implantation in food producing species, large animal models, such as pigs and ruminants, including cows, offer an alternative perspective, particularly for non-invasive studies and for elucidating mechanisms of maternal recognition of pregnancy (Davenport et al., 2023). Porcine models are highly informative for studying prolonged conceptus-epithelium interactions and rapid conceptus elongation (Johnson et al., 2025). However, the high cost and limited availability of genetic tools for ungulates can limit their use in mechanistic studies. Direct *in vivo* investigation of human implantation is constrained by significant ethical and technical challenges. Access to peri-implantation tissues is rare, so most observations rely on indirect clinical outcomes. *Ex vivo* methods, such as culturing endometrial biopsies or explant tissues, preserve cellular diversity and tissue structure (Gellersen and Brosens, 2014). However, these systems are short-lived, highly variable between donors, and unsuitable for long-term studies.

Taken together, traditional models have provided essential insights but offer an incomplete view of implantation biology. 2D systems lack structural and mechanical accuracy, animal models are limited by interspecies differences, and *ex vivo* tissues are constrained by their lifespan. These combined limitations have prompted the development of bioengineered 3D *in vitro* models, such as organoids, assembloids, blastoids, and advanced microfluidic cultures, which combine cellular complexity with physiologically relevant ECM architecture and fluid flow to better mimic the implantation environment.

## Three-dimensional *in vitro* culture systems and models of implantation

Recent advances in stem cell biology, biomaterials, and microengineering have driven the development of sophisticated culture models that closely recapitulate native tissue architecture. These 3D *in vitro* culture models are engineered to support cell-matrix interactions, thereby mimicking the physical and chemical conditions observed *in vivo*. In designing these 3D models, the mechanical properties of the matrix scaffold, interstitial fluid flow, and the spatial arrangement of cells collectively determine biological outcomes (Costa et al., 2016).

These platforms have advanced fundamental biological research, improved disease diagnostics, and supported the development and testing of novel therapeutics (Jensen and Teng, 2020). In the field of implantation, these advanced systems have enabled the development of models that capture crucial interactions between the embryo and the uterine endometrium. These models include spheroid cultures, endometrial and trophoblast organoids (bovine, ovine, and porcine), blastoids, post-implantation models, and advanced microfluidic cultures or organ-on-a-chip systems that incorporate fluid flow to reproduce shear stress and other biophysical cues found in tissues (Figure 1, Tables 1 and 2) (Gnecco et al., 2017; Heidari Khoei et al., 2023; Turco et al., 2018). Crucially, these models emphasise the importance of selecting the appropriate matrix scaffold composition. By mimicking the native ECM architecture and its inherent structural and mechanical properties, these 3D systems enable cells to sense and respond to biophysical cues absent in traditional 2D cultures. To develop practical models of implantation across different mammalian species, it is essential to recapitulate the tissue dynamics of both the endometrium and the embryo/conceptus within a 3D structure that enables investigation of the requirements to support pregnancy at this critical time point.

**Table 1:** Examples of 3D *in vitro* models for embryo implantation studies across species.

3D in vitro models	Description	Species	Purpose of model	References
Endometrial organoids 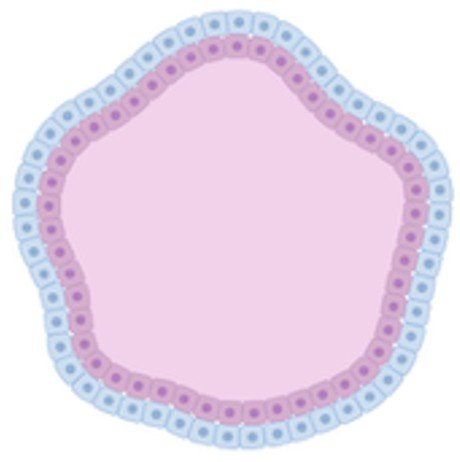	3D cultures of primary epithelial/glandular endometrial cells embedded in a hydrogel (*e.g.,* Matrigel).	Ruminants (*e.g.,* Cows and Sheep) Pig Human Mouse	- Model maternal recognition (IFNτ or E_2_ signals).	Boretto et al. (2017), Edge et al. (2026), Fujimura et al. (2025), Guo, et al. (2026b), Turco et al. (2018)
			- Study the histotrophic composition/glandular secretions.	
			- Test hormone response and Investigate patient-specific drug responses.	
	Replicates apico-basal polarity and secretory functions.			
Blastoids and Assembloids 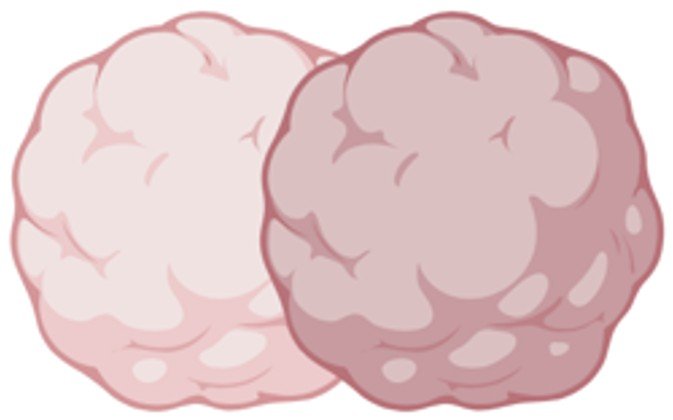	Stem-cell derived to mimic embryo morphology and gene expression:	Cow	- Study maternal-foetal dialogue of implantation.	Munsie and Findlay (2025), Pinzon-Arteaga et al. (2023), Rawlings et al. (2021), Rivron et al. (2018), Xiang et al. (2024)
		Pig		
		Human	- Study early embryonic development or interstitial invasion.	
	- Blastoids: synthetic embryos from stem cells.			
		Mouse	- Model decidualisation triggers.	
			- High throughput screening of genetic and chemical factors affecting embryo attachment.	
	- Assembloids (humans): integration of blastoids and organoids.			
Hydrogel systems 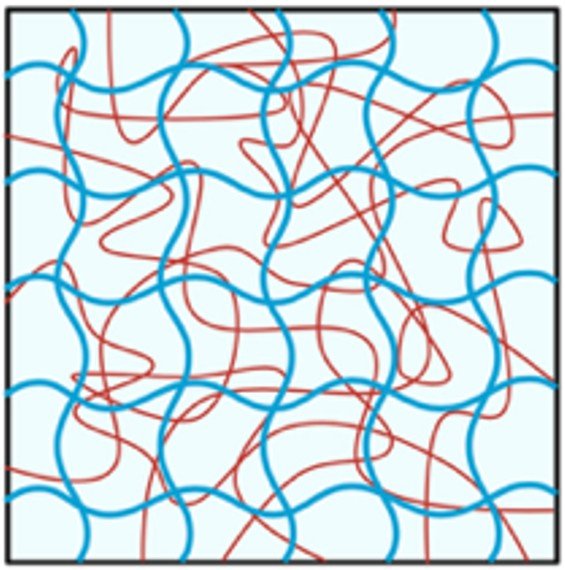	Hydrogel or mix of matrix factors to mimic the extracellular matrix (ECM) environment of the tissue in question.	Cow	- To mimic the ECM environment, preserve layers of the endometrial tissue, and improve organoid culture systems.	Edge et al. (2026), Ichikawa et al. (2022), Lopez-Martinez et al. (2021), Nie et al. (2023), Wang et al. (2021)
		Pig		
		Human		
		Mouse	- To support and reproductive outcomes after surgery and improving endometrial tissue fibrosis.	
	Hydrogels include Matrigel, collagen/GelMA, hyaluronic acid, alginate, and bioprinting scaffolds.			
Organ-on-a-chip 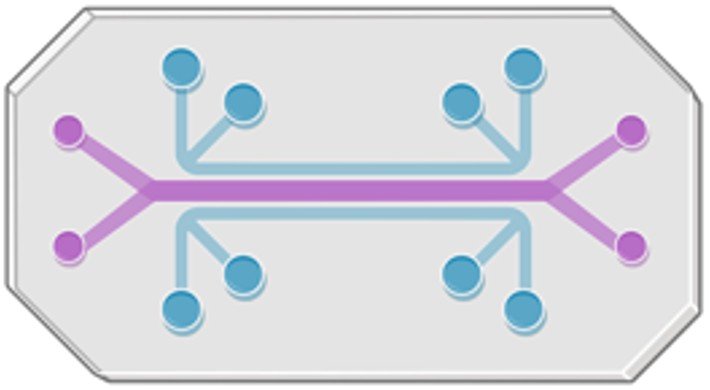	A 3D *in vitro* culture platform that integrates the co-culture of primary derived cells or commercial cell lines, and hydrogel to cell-cell and cell-matrix interactions and the fluid dynamic environment as seen in tissues.	Cows	- To mimic and study decidualisation and hormonal changes during menstrual cycle, and response to hormone treatment and that of the menstrual cycle.	Lee et al. (2025), Tinning et al. (2025)
		Human		
			- To study conceptus-derived proteins that modulate mother’s uterine fluid composition to prepare for implantation.	
		Mouse		
			- Model a study endometrial vasculo-angiogenesis.	
			- Model embryo implantation and study embryo receptivity.	
			- Simulating maternal uterine endometrial and embryo interactions and invasion of EVTs.	

**Table 2:** Comparative summary of engineering approaches for 3D *in vitro* implantation models that can be applied to mammal species.

Engineering approach	Key structural and biological characteristics	Major advantage/strength	Technical limitations/challenges	Examples of applications
Hydrogels and scaffolds (*e.g.,* Matrigel, Alginate, Fibrin-Agarose, dECM, GelMA) 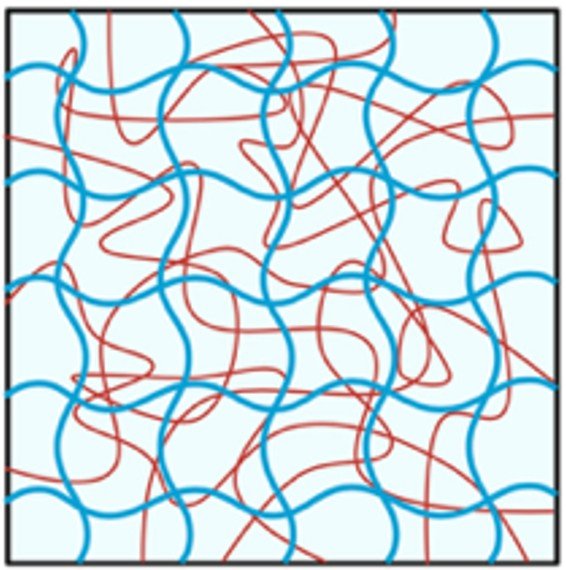	- Complex protein matrices, synthetic polymers, or organ-specific tissue extracts.	- Promote epithelial polarisation and lineage differentiation.	- Batch-to-batch variability in natural extracts.	- Co-culturing endometrial spheroids with extravillous trophoblasts.
			- Undefined bioactive molecules.	
		- Captures layered, multicellular architectures, and epithelial-stromal crosstalk.		
				- Blastocyst embedding, testing matrix stress relaxation, modelling matrix penetration, establishing stable microvascular co-cultures, and testing cell responses to biochemical/topographical cues.
			- Structural degradation can limit long term culture stability.	
	- Tunable viscoelastic and mechanical properties, and degradability profiles.			
			- Static designs face diffusion limits and lack dynamic fluid forces.	
		- Soluble matrices actively supply metabolites to developing embryos.		
3D bioprinted hydrogel constructs 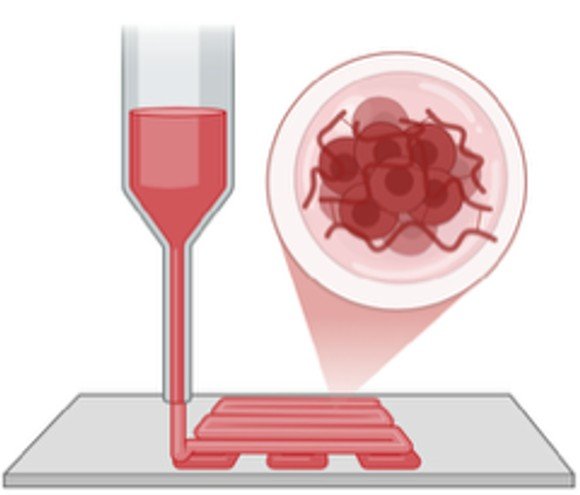	- Automated computer guided spatial deposition of cell-laden hydrogels.	- Enables architectural reproducibility.	- Hydrogel encapsulation density can restrict direct cell-to-cell contact if dense.	- Fabricating hormone-responsive epithelial-stromal bilayer tissue constructs.
		- Precise spatial control over cell types and signalling cues.		
			- Photocrosslinking or shear stress during extrusion can induce cellular stress.	
				- Printing artificial maternal spiral arterioles to drive directional trophoblast migration, and
	- Engineered to construct defined multi-layered layouts or configurations.	- Facilitates the fabrication of localised, directional chemotactic gradients.		
				- enrichment of bio-inks with decellularised tissue components.
Organ-on-a-chip 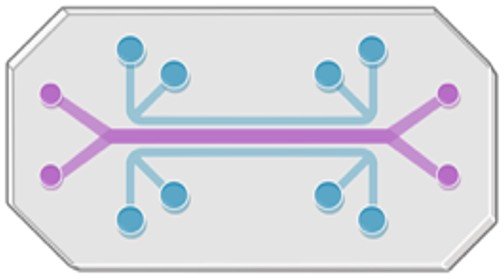	- Perfused microscale fluid channels dividing cellular chambers using porous membranes or hydrogels.	- Successfully replicates interstitial flow, fluid shear stress, and active nutrient exchange.	- High technical complexity to fabricate and standardise.	- Real-time imaging of EVT-maternal vasculature interactions under flow conditions.
		- Generates temporally and spatially defined hormone or hypoxia gradients.		
				- Modelling the placental barrier structure, testing response to dNK cell factors, studying hypoxic early environments, and transitioning patient-derived organoids into stable, polarised monolayers.
			- Current cultures lack underlying stroma or rely heavily on immortalised lines.	
		- Allows real-time visualisation of cellular invasion.		
	- Allows continuous perfusion and dynamic flow.			
		- Precise spatial control over cell types and signalling cues.		

## Endometrial organoids

Endometrial organoids represent a significant advance in *in vitro* models of the endometrium, although they still lack the full complexity of *in vivo* tissue and show species-specific limitations on the number of passages they can undergo. Gland-like endometrial epithelial organoids have now been established and characterised across multiple mammalian species, including human, bovine, ovine, porcine, and murine (Boretto et al., 2017; Edge et al., 2026; Guo et al., 2026b; Saadeldin et al., 2024; Turco et al., 2017). These organoids are derived from primary endometrial tissue, cultured within a supportive extracellular matrix, and grow into hollow, apical-in spheroids that mimic the morphology and secretory function of endometrial glands (Figure 1). They have been used to investigate gland-specific transcriptional responses to hormonal (Turco et al., 2017) and conceptus-derived signals (Edge et al., 2026), and to mimic native polarised tissue architectures by expressing lineage markers such as KRT18 (Guo et al., 2026b), offering insights not easily achieved with traditional 2D culture or *in vivo* models. Organoid methodologies developed in conventional model species are now being applied to more exotic species such as rhinoceros, with early success in generating rhinoceros endometrial organoids (Thompson-Brandhagen et al., 2026), highlighting their potential to advance reproductive research in endangered species. Recently, human apical-out endometrial glandular organoids have been produced (Ahmad et al., 2024; Fujimura et al., 2025; Shibata et al., 2024), providing a gland-like *in vitro* system with direct access to the endometrial glandular secretions that nourish and support early embryo development. Although apical-out endometrial organoids may contain stromal or endothelial cells (Guo et al., 2026b; Fujimura et al., 2025; Shibata et al., 2024), the models remain focused on epithelial function and still lack the complexity of cellular interactions *in vivo*.

To overcome these limitations, endometrial assembloids were first developed (Rawlings et al., 2021) by combining glandular epithelium and stromal cells, and they demonstrated transcriptional and architectural similarities to *in vivo* tissue. More recent human assembloid models incorporate the luminal epithelium using air-liquid interface (ALI) systems (Tian et al., 2023), thereby expanding their potential for implantation studies, where luminal epithelial cells are required for attachment and invasion (Douglas et al., 2026). Endometrial assembloids have also been described in mice (Qiu et al., 2026), combining luminal and glandular epithelium with stromal compartments and more effectively replicating endometrial architecture than organoids. Developing endometrial assembloids in additional mammalian species will further enable studies of multicellular communication and the processes underlying the diverse implantation strategies seen across mammals. Simultaneously, apical-out mouse (Fujimura et al., 2025) and human (Shibata et al., 2024) endometrial organoids have been co-cultured with blastocysts and or blastoids, demonstrating their ability to support attachment, epithelial breaching, and invasion, thus mimicking key aspects of the *in vivo* implantation process in these species.

Together, these advancements highlight the potential to develop increasingly advanced integrated, whole-tissue, multicellular *in vitro* implantation systems, as recently reviewed (Rawlings et al., 2026). Endometrial organoids and assembloids offer powerful platforms for investigating endometrial roles in early pregnancy and complement other techniques. Ongoing development of advanced *in vitro* models, including the integration of engineering approaches, will further improve our ability to model and understand the complex processes governing embryo implantation.

## Embryo models

In food production species such as ruminants, access to *in vitro* produced embryos is relatively straightforward up to the blastocyst stage. However, because these embryos undergo conceptus elongation before implantation (including in pigs, cows, and sheep), true *in vitro* conceptus elongation has not yet been achieved despite multiple attempts (Brandão et al., 2004; Ramos-Ibeas et al., 2020). In these species, *in vitro* implantation studies remain limited to systems that support post-hatching embryo attachment without elongation (Ramos-Ibeas et al., 2022) or rely on specialised embryo models. In other species, access to mature oocytes can be challenging (*e.g.,* humans) and is limited in availability.

A relatively accessible and widely used method employs trophoblast spheroids. Human trophoblast cell lines such as BeWo, JEG-3, or JAR have been used to generate simple, embryo-sized, dense, spherical spheroids (*i.e.,* without a lumen) by aggregation. These spheroids can then be co-cultured with human endometrial cells to examine attachment and or invasion, although each cell line has functional limitations (Hannan et al., 2010). Alternatively, inert embryo beads or similarly sized beads can be used to assess specific receptor-ligand interactions during endometrial attachment (Kang et al., 2014).

Trophoblast organoids offer a more physiologically relevant alternative to trophoblast spheroids, as they are derived from primary trophectoderm or trophoblast stem cells and thus model the trophoblast lineages that interact with the endometrium during implantation and subsequently form the placenta. Human trophoblast organoids have been characterised from first-trimester trophoblast stem cells (Haider et al., 2018; Turco et al., 2018) and have been shown to differentiate into various placental lineages, including EVTs and syncytiotrophoblasts. Mouse trophoblast organoids have also been developed and contain cell populations undergoing placental lineage differentiation (Mao et al., 2023). Porcine trophoblast organoids have been developed and characterised from term placenta (McCutcheon et al., 2025), while bovine trophoblast organoids have been formed from 3 month gestation placenta and immortalised trophoblast cell lines (Liu et al., 2024). However, because these ungulate trophoblast organoids are derived from later stage, differentiated trophoblasts, they may not accurately represent the peri-implantation trophectoderm in ungulates, limiting ungulate studies to trophoblast spheroids or *in vivo* studies. This highlights the need for further development in this area for domestic species.

A further step towards physiological relevance is the use of blastoids (blastocyst-like structures) derived from pluripotent stem cells. These are typically generated from primed embryonic stem cells (ESCs) and induced pluripotent stem cells (iPSCs), as well as from expanded or extended pluripotent stem cells (EPSCs). Production relies on specialised 3D culture techniques, including aggressive aggregation in non-adhesive microwells, microfluidic cell-trapping platforms, and the precise, sequential application of growth factor and small-molecule cocktails that drive multi-lineage self-organisation (Liu et al., 2024). Unlike models that include only trophectoderm, blastoids feature epiblast-like, hypoblast-like, and trophoblast-like lineages, which better mimic the cellular architecture of the blastocyst stage embryo and have been produced to model human (Kagawa et al., 2022; Karvas et al., 2023), murine (Li et al., 2019; Mao et al., 2023), and bovine (Pinzon-Arteaga et al., 2023) blastocyst formation and implantation. Blastoids offer several additional benefits, including reduced reliance on embryo sourcing and increased experimental throughput. They can also be integrated with microfluidic or organ-on-a-chip platforms (Li et al., 2026), creating a more advanced system for modelling implantation in a controlled *in vitro* environment.

## Engineering approaches for three-dimensional *in vitro* implantation models

### Three-dimensional scaffold-based models of embryo implantation

Standard 2D models are highly valuable for high-throughput screening. Moreover, advanced 2D platforms, such as ALI systems, successfully achieve robust epithelial cell differentiation, apical-basal polarisation, and functional tight junctions. However, these systems remain limited because they lack an expansive 3D mechanical context and complex spatial signalling gradients. Implantation requires a blastocyst to navigate multi-directional morphogen gradients diffusing through a surrounding extracellular matrix, a phenomenon that flat, planar surfaces cannot replicate. Furthermore, 2D models fail to capture crucial 3D physical cues, such as interstitial fluid shear stresses, circumferential stretch, and the varied mechanical resistance of deep stromal tissue. Consequently, while differentiated 2D barriers are highly reliable for initial adhesion assays, 3D scaffold systems address these shortcomings by embedding cells within structurally defined microenvironments that more closely resemble native tissue. Importantly, the scaffold itself is not an inert support. Its stiffness, viscoelasticity, degradability, and adhesion ligand profile actively regulate cell proliferation, migration, differentiation, and gene expression. In addition, 3D microfluidic and organ-on-a-chip platforms are necessary to truly mimic the structural deformation and invasive signalling dynamics of the embryo-maternal interface.

Information on the mechanical properties of the endometrium in mammalian species is limited, particularly in food production species. In humans, these properties vary both spatially and temporally across the menstrual cycle and during the implantation window (Abbas et al., 2019). Various biomaterials have also been developed for endometrial regeneration and repair, and their potential applications in implantation modelling warrant further research. However, these systems are beyond the scope of this review and have been discussed in the literature elsewhere (Cadena et al., 2021; Kim and Lee, 2026). This section discusses scaffold platforms used directly in embryo implantation and early embryonic development, organised by model type. A comprehensive comparison of these hydrogel matrices, bioprinted systems, and advanced microfluidic platforms is presented in Table 2.

### Macro-scale three-dimensional tissue constructs

Macro-scale constructs aim to replicate the endometrium’s layered cellular architecture at the tissue level. Basement membrane extracts such as Matrigel and Cultrex were among the earliest matrices used for 3D endometrial organoid development (Boretto et al., 2017; Edge et al., 2026; Saadeldin et al., 2024; Turco et al., 2017). Their complex protein composition, including laminin, collagen IV, entactin, and heparan sulphate proteoglycan, provides a suitable environment for cell culture. Buck et al. (2015) co-cultured human endometrial epithelial spheroids with extravillous trophoblast cells within Matrigel to investigate how glandular epithelial polarisation influences trophoblast invasiveness (Buck et al., 2015). Chang et al. (2018) embedded primate blastocysts in Matrigel supplemented with feeder-conditioned medium from Buffalo Liver Rat cells, enabling the study of trophoblast invasion and lineage marker expression beyond what is possible in planar cultures (Chang et al., 2018). Recently, Guo et al. (2026a) tuned the Matrigel concentration to match the viscoelastic properties of the decidua and demonstrated that 3D embryo culture is more effective than 2D culture for developmental success. Furthermore, they employed chemical crosslinking to modify Matrigel characteristics and showed that matrix stress relaxation and degradability influence focal adhesion formation at the trophoblast–gel interface, facilitating the matrix remodelling needed for embryo growth to an early organogenesis-like stage (Guo et al., 2026a). Despite these applications, these matrices, including Matrigel, have recognised drawbacks. Batch-to-batch variation in growth factor and protein content hinders reproducibility, undefined bioactive molecules complicate mechanistic interpretation, and matrix properties such as stiffness and degradability cannot be tuned independently of their biochemomposition. These limitations have led to the adoption of alternative natural and synthetic biomaterials with greater compositional control.

Alginate has been widely used for embryo encapsulation owing to its biocompatibility and tunable gelation chemistry. Zhao et al. (2015) demonstrated that culturing post-hatched bovine embryos within alginate beads for up to 18 days resulted in superior survival, expansion, and elongation compared with standard culture, with morphological changes mirroring those observed *in vivo*. This suggests the necessity of a 3D matrix to provide appropriate *in vitro* culture conditions. In this study, they detected binucleate-like cells expressing pregnancy-associated glycoprotein after culturing retrieved embryos in the 3D culture model (Zhao et al., 2015). Sargus-Patino et al. (2013) similarly employed alginate encapsulation to support porcine blastocyst elongation *in vitro*, maintaining viability and structure while enabling steroidogenic gene expression and oestrogen production.

Gao et al. (2023) expanded this approach by engineering alginate microcarriers loaded with mouse trophoblast stem cells as blastocyst models, where laminin surface modification provided adhesion sites and increased carrier stiffness, significantly shifting the transcriptomic profile of loaded cells towards stemness-associated gene expression and promoting deeper invasion and a larger outgrowth area in implantation experiments (Gao et al., 2023). To mimic endometrial tissue structure, Catane et al. (2025) used an alginate-based bio-ink to bioprint a bilayer construct encapsulating human epithelial and stromal cell lines. The construct proved hormone-responsive and supported trophoblast spheroid adhesion and infiltration, serving as an implantation model (Catane et al., 2025).

Fibrin-agarose composites were used by Wang et al. (2012) to develop a stratified endometrial model in which stromal cells were embedded in the matrix and epithelial cells formed a surface monolayer. This architecture enabled investigation of epithelial-stromal crosstalk under hormonal stimulation with 17β-estradiol (E2) and medroxyprogesterone acetate (MPA), as well as assessment of human trophoblast (JAR) cell spheroid attachment and invasion, thereby capturing multicellular implantation dynamics within a physiologically layered environment (Wang et al., 2012, 2013).

One major approach has been to investigate the use of decellularised extracellular matrix (dECM) hydrogels to provide organ-specific biochemical complexity while removing cellular immunogens. Francés-Herrero et al. (2021) developed a rabbit oviductal dECM (oviECM) hydrogel to support early embryo development. Proteomic analysis showed high collagen content alongside reduced glycosaminoglycan (GAG) levels following decellularisation. When 2-cell rabbit embryos were cultured on oviECM-coated surfaces, blastocyst development rates and embryo morphology were comparable to those under standard culture conditions. However, metabolomic analysis of conditioned media revealed that the oviECM scaffold actively contributed amino acids and glycolytic intermediates to the culture environment, thereby alleviating the metabolic burden on the embryos and freeing resources for development (Frances-Herrero et al., 2021). Crucially, this early metabolic priming and optimisation during oviductal transit are vital determinants of embryo quality, directly affecting the embryo’s subsequent capacity for successful attachment and invasive implantation within the uterine endometrium. López-Martínez et al. (2021) produced porcine uterine dECM scaffolds and demonstrated that endometrial cells proliferated more extensively within them than in collagen or Matrigel controls, although the gel's high degradability limited long-term culture stability (Lopez-Martinez et al., 2021). Sadeghi et al. (2023) decellularised human endometrial ECM with human endometrial mesenchymal cells and implanted mouse embryos onto the reconstituted tissue; embryo attachment and matrix penetration occurred within 48 hours, accompanied by significantly elevated expression of decidualisation markers, including SSP1, matrix metalloproteinases (MMP) 2, and PRL, and increased β-hCG and prolactin secretion compared to the control group without embryos, confirming the bioactivity of the human ECM in driving implantation-related processes (Sadeghi et al., 2023).

Gelatin methacryloyl (GelMA) has become one of the most widely used biomaterials in this field, combining inherent biological activity, such as cell adhesion motifs and protease-cleavable sequences from its gelatin origin, with photocrosslinkable tunability that allows stiffness to be adjusted independently by varying the polymer concentration, photo-initiator levels, and light exposure. Zambuto et al. (2019) fabricated GelMA constructs with mechanical properties matching those of native endometrial tissue and validated their ability to support epithelial monolayer culture (depending on cell seeding density and culture duration), HUVEC-stromal co-culture for endometrial angiogenesis modelling, and trophoblast spheroid invasion assays on a single platform (Zambuto et al., 2019). Subsequent work by the same group used decidua-matched GelMA to encapsulate HTR-8/SVneo trophoblast spheroids and measure invasion responses to molecular stimuli. EGF increased motility and resulted in a coronal invasion pattern, TGF-β1 inhibited outgrowth while maintaining a thorn-like morphology, and cortisol showed no measurable effect (Zambuto et al., 2021). Zambuto et al. (2022) further applied GelMA encapsulation to show that pregnancy-specific glycoproteins (the most abundant circulating trophoblastic proteins in maternal blood during human pregnancy) differentially regulate trophoblast motility. PSG9 decreased outgrowth, and PSG1 increased it, alongside characterisation of responses to EGF (an invasion promoter) and Nodal (an invasion inhibitor) (Zambuto et al., 2022). Recently, they used GelMA to develop stable endometrial microvascular networks with an optimised 2:1 human microvascular endothelial-stromal co-culture ratio. They also reported that decidualisation changed the microvascular network's complexity by increasing the total number of vessels and altering the secretome composition, while the overall network length and branch number varied depending on the specific decidualisation protocol used. Furthermore, conditioned medium from decidualised networks reduced trophoblast motility. Incorporating an epithelial monolayer into this system created a tri-culture construct that better mimics the complexity of the endometrium (Zambuto et al., 2024).

GelMA has also been the primary bio-ink for bioprinted implantation models featuring defined spatial gradients. Kuo et al. (2018) printed a multi-layered cylindrical structure with an EGF-laden GelMA core resembling a maternal spiral arteriole and establishing a radial chemotactic gradient, encased by an outer layer of BeWo trophoblast cells, thereby demonstrating dose-dependent EGF-driven migration. Epidermal growth factor (EGF) is a key signalling molecule involved in decidualisation, implantation, and trophoblast invasion (Kuo et al., 2016). They enriched the bio-ink with proteins from decellularised human term placenta and, using proteomics, confirmed the presence of laminin, collagen, fibronectin, heparan sulphate proteoglycan, and nidogen. They also demonstrated that this basement membrane environment enhanced invasion rates and increased MMP2 and MMP9 expression, linking ECM composition to trophoblast invasiveness and preeclampsia-related phenotypes (Kuo et al., 2018). Ding et al. (2019) used a similar multi-ring GelMA structure with a trophoblast outer ring to assess EGF's stimulatory effects on HTR-8/SVneo migration (Ding et al., 2019). Kuo et al. (2019) further developed this platform by incorporating a perfusion bioreactor to introduce interstitial fluid shear stress and facilitate the study of trophoblast-endothelial interactions under dynamic conditions (Kuo et al., 2019).

Among the most complex macro-scale systems reported, Molè et al. (2026) developed the CREST platform to advance our understanding of early human embryo implantation. The model reconstructs the superficial layer of the human receptive endometrium by integrating the luminal epithelial, glandular, and stromal compartments into a single co-culture system. A dextran-based hydrogel (TrueGel3D), supplemented with Collagen I, III, and VI and Fibronectin, was used to mimic the endometrial extracellular matrix and provide adhesion sites for encapsulating stromal cells. Epithelial organoid fragments, containing progenitor cells capable of differentiating into both luminal and glandular epithelial subtypes, were seeded onto the stromal hydrogel. The resulting co-culture was subjected to sequential hormonal stimulation to mirror the proliferative and mid-secretory phases of the menstrual cycle. The model demonstrated hormone responsiveness, undergoing decidualisation as evidenced by morphological remodelling in both compartments, increased secretion of prolactin and IGFBP1 by stromal cells, altered epithelial expression of ciliary and glandular markers, and elevated glycodelin (PAEP) levels. Gland-like cavities were also observed within the epithelial layer. Fully expanded blastocysts and blastoids derived from the naïve human embryonic stem cell line HNES1 were subsequently transferred onto the endometrial scaffolds. Compared with acellular 2D and 3D controls, the presence of endometrial cells significantly improved both attachment efficiency and the spatial organisation of primary embryonic lineages during post-implantation development. Key post-implantation structures were evident by days 12 and 14, including an organised epiblast, yolk sac cavity, putative amniotic cells, and extraembryonic mesoderm. Furthermore, the model enabled transcriptomic characterisation of the implantation niche. Despite these advances, the platform currently faces limitations due to a scaffold thickness of approximately 180 µm, which prevents modelling of deep glandular structures and trophoblast invasion, and it lacks vascular and immune cell compartments, restricting its broader physiological relevance and high-throughput capability (Molè et al., 2026).

Beyond hydrogel-based systems, Guo et al. (2022) fabricated anisotropic microporous PDMS scaffolds with varying angles of structural anisotropy to produce surfaces with different porosity, wettability, and contact geometry. They showed that topographical cues alone, independent of biochemistry, shaped the spreading areas and developmental behaviour of mouse embryos 61. It is clear that using dECM can increase the complexity of the 3D systems required to study implantation events in mammals (Guo et al., 2022).

### Advanced microfluidic cultures or organ-on-a-chip (OoC) models of implantation

Microfluidic technology provides a powerful platform for developing physiologically relevant 3D *in vitro* models by enabling precise control over the biochemical and mechanical cellular landscape (Meyvantsson and Beebe, 2008). Microscale fluid channels enable continuous perfusion, precise spatial compartmentalisation of cells, and regulation of molecular gradients. This allows organ-on-chip systems to mimic *in vivo* fluid dynamics and nutrient exchange accurately replicates *in vivo* morphology and pathophysiology in a reproducible, scalable, and high-throughput manner (Leung et al., 2022; Meyvantsson and Beebe, 2008). It also enables the creation of temporally and spatially defined gradients of hormones and cytokines, which, in the context of implantation, allow modelling of endocrine fluctuations across the oestrus/menstrual cycle and of localised embryo-derived effects (Gnecco et al., 2023).

Although the uterine environment is not characterised by high fluid shear, subtle mechanical forces and tissue deformation associated with fluid flow influence epithelial polarity, trophoblast adhesion, and invasion. This was shown by Park et al. (2022), who developed a microfluidic model of the human implantation environment with integrated vasculature to observe trophoblast-directed angiogenesis and nutrient exchange under physiological flow conditions (Park et al., 2022). In this device, trophoblasts were embedded within a collagen type I matrix adjacent to perfused endothelial channels, allowing real-time imaging of EVT invasion and interactions with the engineered maternal vasculature. Using primary human EVTs, decidual stromal cells, uNK cells, and human microvascular endothelial cells, they demonstrated that trophoblasts migrated towards endothelial structures, accompanied by active endothelial remodelling. This process is challenging to capture with static culture systems or animal models. Importantly, they showed that decidualised stromal and uNK cells help regulate trophoblast invasion dynamics, highlighting how microengineering can extend beyond simple co-culture to reveal mechanotransduction pathways governing the early maternal-foetal interface. By integrating complex fluid dynamics and spatial compartmentalisation, this approach highlighted the importance of multicellular crosstalk and the microenvironment for human-specific insights.

Prior to this, placenta-on-a-chip cultures have been developed to study aspects of maternal-foetal interaction under dynamic flow conditions. For example, Pu et al. (2021) developed a 3D PDMS-based microfluidic device using gelatin, Matrigel, and fibronectin hydrogels for an ECM rich perfusion culture of the human trophoblast cell line CRL-3271 and HUVEC cells (Pu et al., 2021). This enabled real-time monitoring of trophoblast invasion under flow and allowed assessment of how ECM composition and co-culture interactions influence invasion depth and morphology. Compared with 2D invasion assays, the system provided improved spatial control and facilitated downstream recovery and analysis of invasive cells.

Similarly, Abbas et al. (2017) developed a three-channel microfluidic device to model the maternal-decidua-trophoblast interface. They investigated how maternal decidual natural killer (dNK) cell derived granulocyte-macrophage colony-stimulating factor (GM-CSF) influences EVT migration. They demonstrated directional migration of EVTs towards GM-CSF and showed that activating the dNK cell receptor KIR2DS1 increases GM-CSF production, thereby enhancing EVT invasiveness. This mechanistic insight aligns with *in vivo* data and enables real-time, controllable modulation.

Other platforms have focused on modelling the structure and transport function of the placental barrier. Blundell et al. (2016) developed a two-chamber device for co-culturing BeWo trophoblast cells and human placental villous endothelial cells (HPVECs), separated by a thin polycarbonate membrane (Blundell et al., 2016). Under medium shear stress, the system promoted the formation of dense, villous-like microvilli and trophoblast syncytialisation (with forskolin in the medium), increased secretion of the trophoblast differentiation marker β-hCG, and enhanced glucose transport via glucose transporter 1 (GLUT1), thereby mimicking *ex vivo* placental function. Similarly, Lee et al. (2016) developed a two-channel ECM-based device for co-culturing JEG-3 trophoblasts and HUVECs, separated by a thin ECM layer (Lee et al., 2016). The model maintained confluent epithelial and endothelial layers and showed increased glucose transport via GLUT1 and metabolic activity consistent with *in vivo* observations.

Jeong et al. (2024) developed a hypoxia mimicking placenta-on-a-chip platform to investigate how low oxygen levels in the early implantation environment affect trophoblast invasion and vascular remodelling (Jeong et al., 2024). By controlling oxygen levels in the microfluidic co-culture system, they demonstrated that hypoxia increases trophoblast MMP secretion, promoting ECM degradation, and facilitating endothelial remodelling in adjacent HUVEC channels. These findings offer insights into how early gestational oxygen gradients influence placental development and may lead to pathologies such as preeclampsia. However, the model lacks decidual stromal or immune components, limiting its ability to fully mimic the multicellular complexity of the implantation environment.

Shin et al. (2026) developed a microfluidic device coated with collagen type I and fibronectin that transitions patient-derived endometrial organoids into a stable, polarised epithelial monolayer (Shin et al., 2026). The system demonstrated hormone-responsive transcriptional profiles and showed that the endometrial epithelium can maintain its physiological barrier function and apicobasal polarity under continuous perfusion, providing an accessible interface for embryo attachment studies. However, the model lacks an underlying stromal compartment, preventing the study of trophoblast invasion or decidualisation.

Beyond individual microfluidic devices, integrated microphysiological systems have been developed to combine multiple reproductive tract compartments, including the endometrium, vasculature, and ovarian tissue, to mimic cyclic hormone profiles and endocrine feedback loops relevant to implantation and the menstrual cycle (Wang et al., 2025). These multi-organ architectures aim to extend implantation modelling beyond isolated interfaces, enabling the study of implantation within a physiological context of the entire reproductive tract. Furthermore, although most microfluidic implantation studies have focused on human implantation, similar design principles and findings can be applied to other species. For example, Ferraz et al. (2018) co-cultured bovine zygotes with bovine oviduct epithelial cells (BOECs) in a 370 μm deep two-chambered device separated by a porous membrane (Ferraz et al., 2017, 2018) and observed embryos with transcriptomic and global DNA methylation profiles more similar to those of *in vivo* zygotes than to those of conventional IVF counterparts. This emphasises the model’s ability to maintain epigenetic integrity, which is essential for subsequent development. Such systems provide valuable tools for modelling fertilisation and early embryogenesis in livestock, as well as comparative implantation biology.

Altogether, these models have enabled real-time visualisation of trophoblast migration and invasion, improved understanding of stromal decidualisation, and provided insights into placental barrier function, nutrient transport, and the influence of microenvironmental cues, such as hypoxia, on vascular interactions. These insights could not have been replicated with 2D cultures or animal models (Yan et al., 2023), which primarily model post-implantation rather than the initial embryonic attachment. However, these models have established design principles necessary for more advanced, implantation-specific platforms. These studies have emphasised compartmentalising the uterine endometrium and embryo cells using membranes or hydrogels to enable controlled paracrine signalling and directional invasion, implementing dynamic perfusion to overcome the diffusional limitations inherent in static cultures, and tuning ECM composition to mimic the native microenvironment. Furthermore, they highlight the importance of multicellular co-cultures that incorporate stromal, epithelial, endothelial, and immune cell populations to better reflect the complex *in vivo* microenvironment.

### Imaging approaches to looking at three-dimensional *in vitro* implantation models

All the above mammalian implantation models aim to replicate a process that is inherently 3D and multiscale. Conventional 2D imaging typically offers only a snapshot at a single scale, limiting biological interpretation and quantitative modelling of these systems. Therefore, 3D imaging provides a crucial bridge between experimental implantation models and *in vivo* representative computational frameworks, enabling the reconstruction of tissue geometry, cell–cell interfaces, and spatial relationships necessary to parameterise and validate models of implantation dynamics. Importantly, when combined with imaging of native tissue, 3D approaches enable more precise comparisons between *in vitro* models and the *in vivo* implantation environment.

Whole-mount confocal and light-sheet fluorescence microscopy enable volumetric imaging and allow specific cell populations, signalling pathways, or structural features to be visualised using fluorescent reporters, antibodies, or dyes. Fluorescence imaging is inherently hypothesis-driven and probe-dependent, as the structures observed are constrained by a priori assumptions about which molecular targets are relevant. However, it provides an incomplete picture of tissue architecture and may miss unlabelled or unanticipated structural features that influence mechanical interactions or transport processes during implantation. Labelling cells with dyes or expressing fluorescently tagged proteins enables time lapse imaging of how cells divide and migrate to support implantation.

Volume electron microscopy (volume EM) enables 3D reconstruction of tissues at nanometre-scale resolution from serial electron micrographs. In implantation models, this method is particularly effective at revealing cellular and subcellular architecture, including epithelial–trophoblast interfaces, basement membranes, cell junctions, and organelle organisation. Unlike fluorescence microscopy, EM contrast arises from heavy-metal staining of cellular components rather than from predefined molecular probes. Consequently, volume EM offers probe-independent, hypothesis-agnostic structural contrast, providing a comprehensive view of tissue organisation that is particularly powerful for discovery and for testing hypotheses within computational models. The main limitations include field of view, throughput, cost, and the complexity of sample preparation, which generally restrict analysis to selected regions rather than to entire specimens.

X-ray–based techniques, including micro-computed tomography (microCT) and synchrotron CT, occupy a complementary position between light and electron microscopy. Once considered too low in resolution for cellular biology, microCT imaging now permits near-cellular resolution, expanding its applicability to biological systems, including implantation models (Yoneyama et al., 2025). A strength of X-ray imaging is its ability to capture whole-sample geometry non-destructively, serving as an entry point for correlative workflows. For example, synchrotron microCT can map the complete 3D organisation of an embryo interacting with an organoid or uterine tissue, enabling targeted volume EM of regions of interest that would otherwise be difficult to locate (Laundon et al., 2023). Alternatively, correlative X-ray histology enables entire specimens to be imaged by microCT prior to paraffin embedding, allowing conventional histological sections stained with a wide range of dyes to be placed back into a 3D anatomical framework (Laundon et al., 2025). This approach preserves spatial context while retaining the interpretability and flexibility of classical histopathology.

For computational modelling, 3D imaging pipelines enable genuine multi-scale parameterisation, including cell number, identity, and spatial localisation. These features are critical for modelling cell–cell communication mediated by juxtacrine and paracrine signalling. By integrating hypothesis-driven and hypothesis-agnostic datasets, correlative 3D imaging provides a robust foundation for building and validating *in vivo*-relevant models of mammalian implantation. In turn, 3D imaging informed modelling can help relate findings from *in vitro* systems to native tissue organisation.

## Conclusion

The complexity of mammalian implantation poses an intriguing biological question. However, this complexity also limits the ability to test hypotheses and address early pregnancy loss across species. Despite recent advances in reproductive research, current 3D *in vitro* implantation models face new challenges, including standardisation, reproducibility, and biological validation. Furthermore, many existing platforms lack critical components of the *in vivo* implantation niche, such as immune, vascular, and endocrine interactions, limiting their ability to accurately model the native maternal-foetal interface (Fan et al., 2025). Overcoming these barriers requires integrating interdisciplinary approaches and collaboration between reproductive biologists and engineers to bridge this gap and develop the next generation of physiological models. By incorporating the biophysical properties of implantation through ECM and microfluidic techniques, we can develop more manageable and physiologically relevant tools to study implantation. Moreover, advances in single-cell mapping of key tissues across species (Arutyunyan et al., 2023; Marečková et al., 2024; Vento-Tormo et al., 2018; Evans et al., 2020), alongside advances in imaging capabilities, will enable us to use the most suitable model for the hypothesis we are testing.

## Data Availability

No research data were used.
